# Supplementary material for: Large scale plasma proteomics identifies novel proteins and protein networks associated with heart failure development
Source: Nat Commun. 2024 Jan 15;15:528. doi: 10.1038/s41467-023-44680-3 (PMC10789789; doi:10.1038/s41467-023-44680-3)

Supplementary Information

Large scale plasma proteomics identifies novel proteins and protein networks associated with heart failure development

Shah et al.

Table of Contents:
Supplementary Figures 1-10

Supplementary Figure 1: Consort diagram for ARIC participants at study Visit 3.

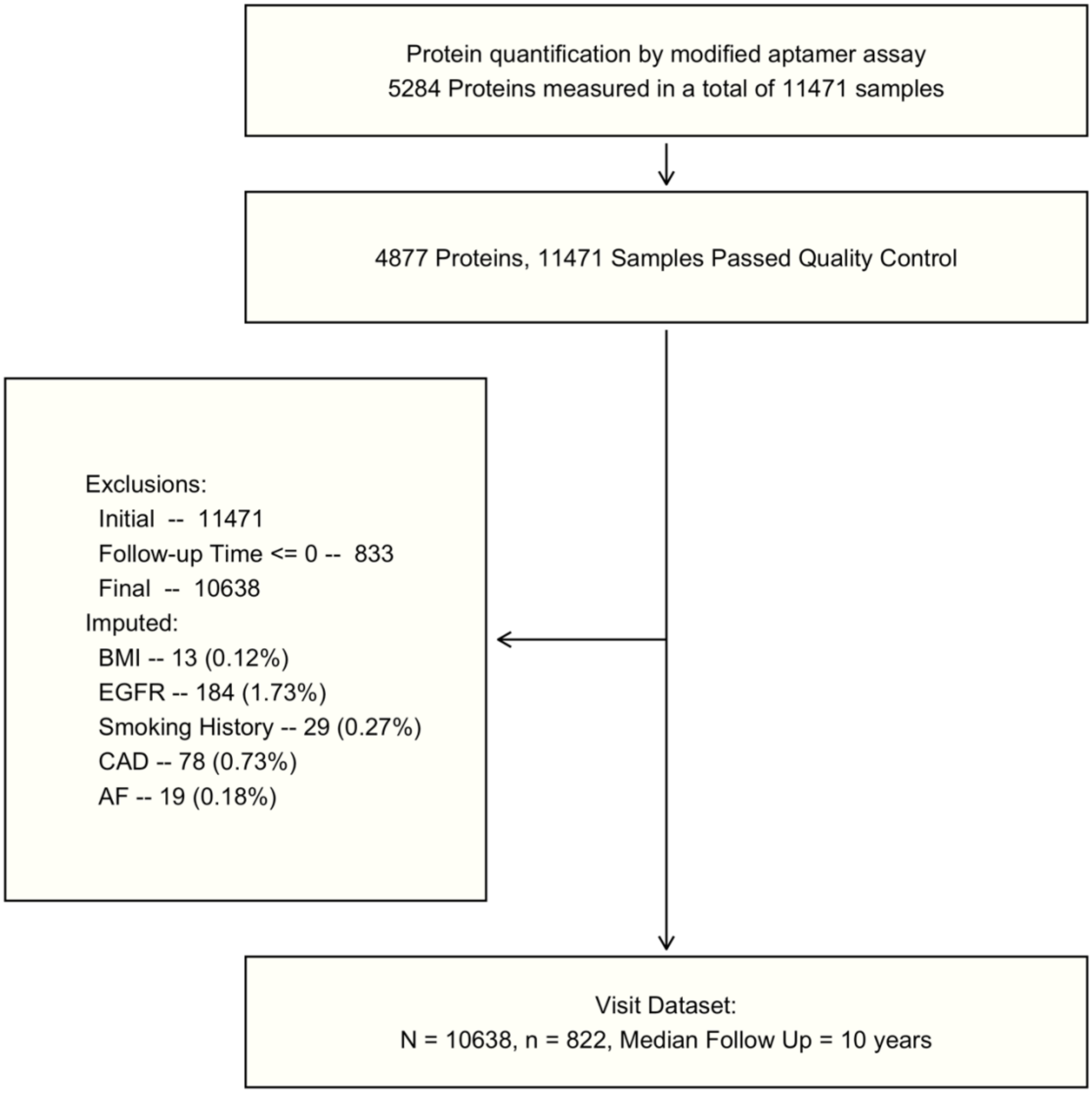

Supplementary Figure 2: Consort diagram for ARIC participants at study Visit 5.

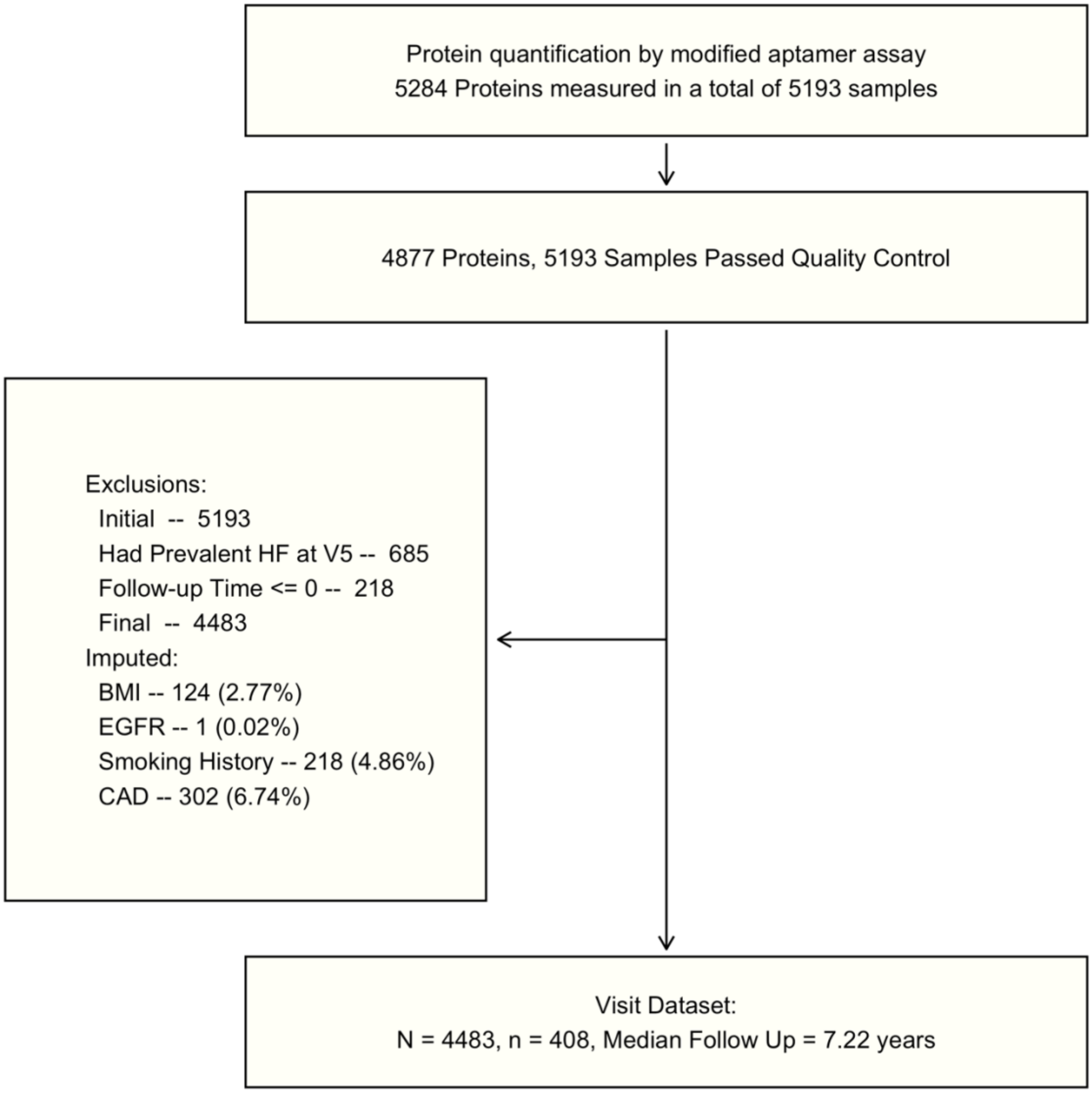

Supplementary Figure 3: Consort diagram for HUNT participants.

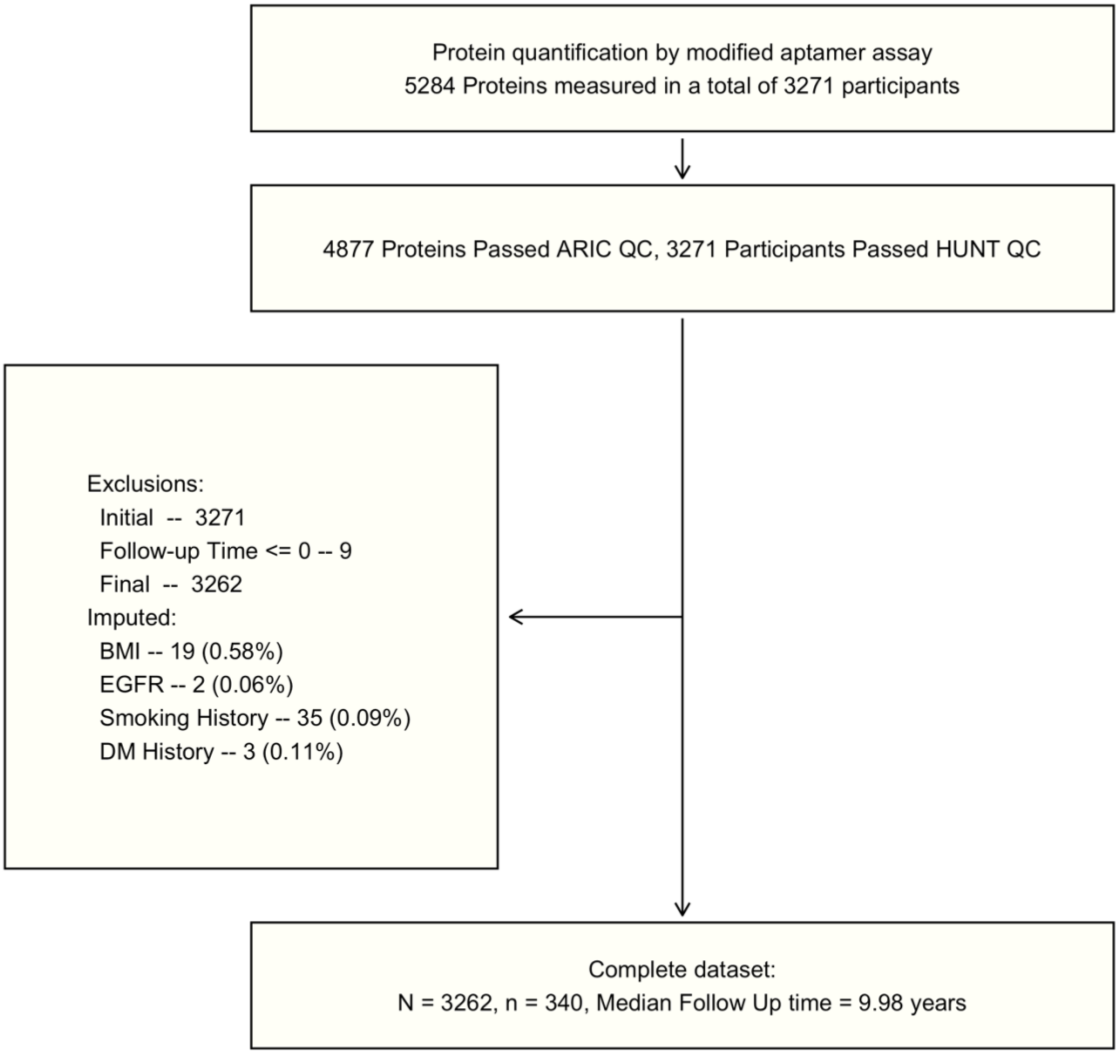

Supplementary Figure 4: Volcano plot of the association of individual plasma proteins with incident HF based on inverse variance meta-analysis of ARIC visit 3 (n= 10,638), ARIC visit 5 (n= 4,483), and HUNT (n=3,262) data. Y-axis lines indicate significance threshold for FDR <0.05 (lower line) and Bonferroni significance (upper line). Red data points represent proteins identified in the primary parallel analysis. Source data are provided as a Source Data file.

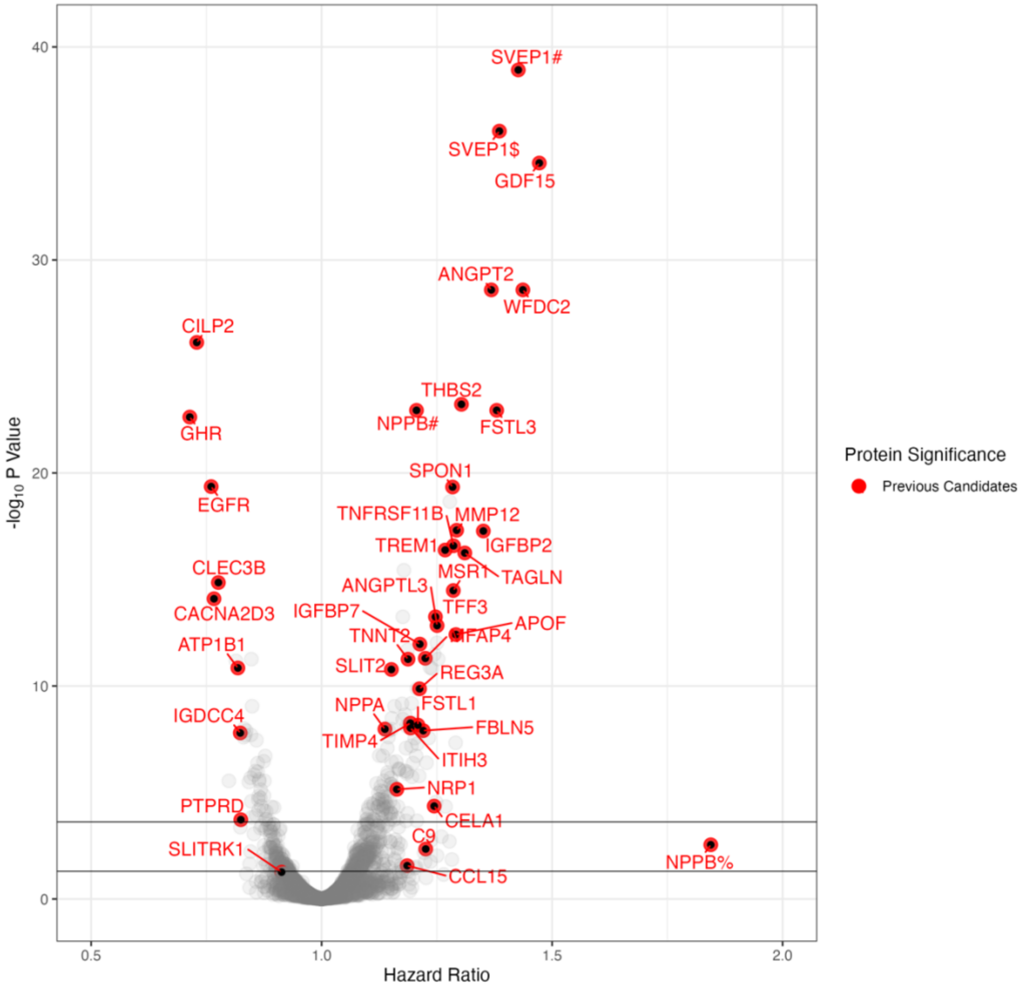

Supplementary Figure 5: Correlation plots of the 39 candidate aptamers at (a) ARIC late-life analysis set; (b) ARIC mid-life analysis set; and (c) HUNT. Source data are provided as a Source Data file.

a.

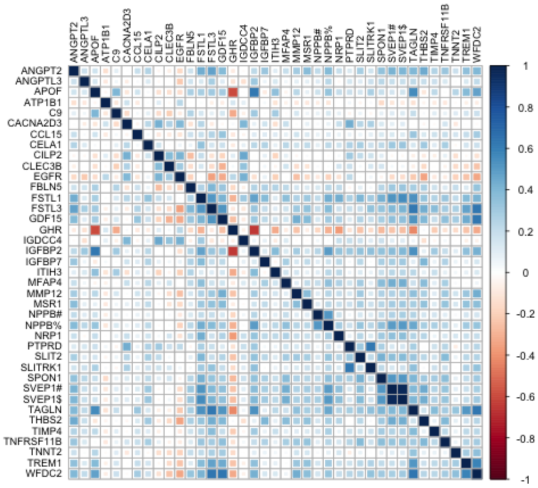

b.

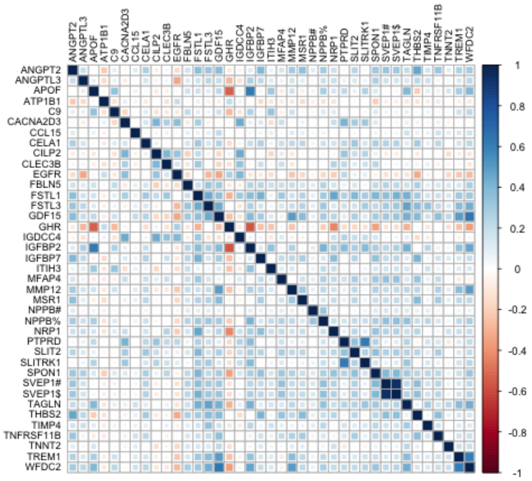

c.

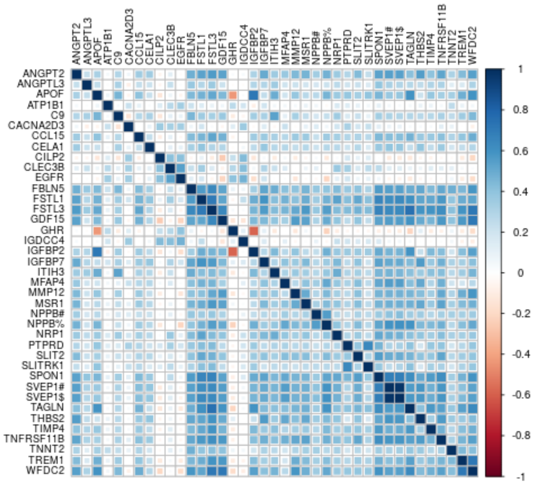

Supplementary Figure 6: Scatter plots and associated Pearson correlations for Somascan aptamer levels (Y-axis) and orthogonal antibody-based assay (X-axis; see Methods). Values from all assays were log2-transformed.

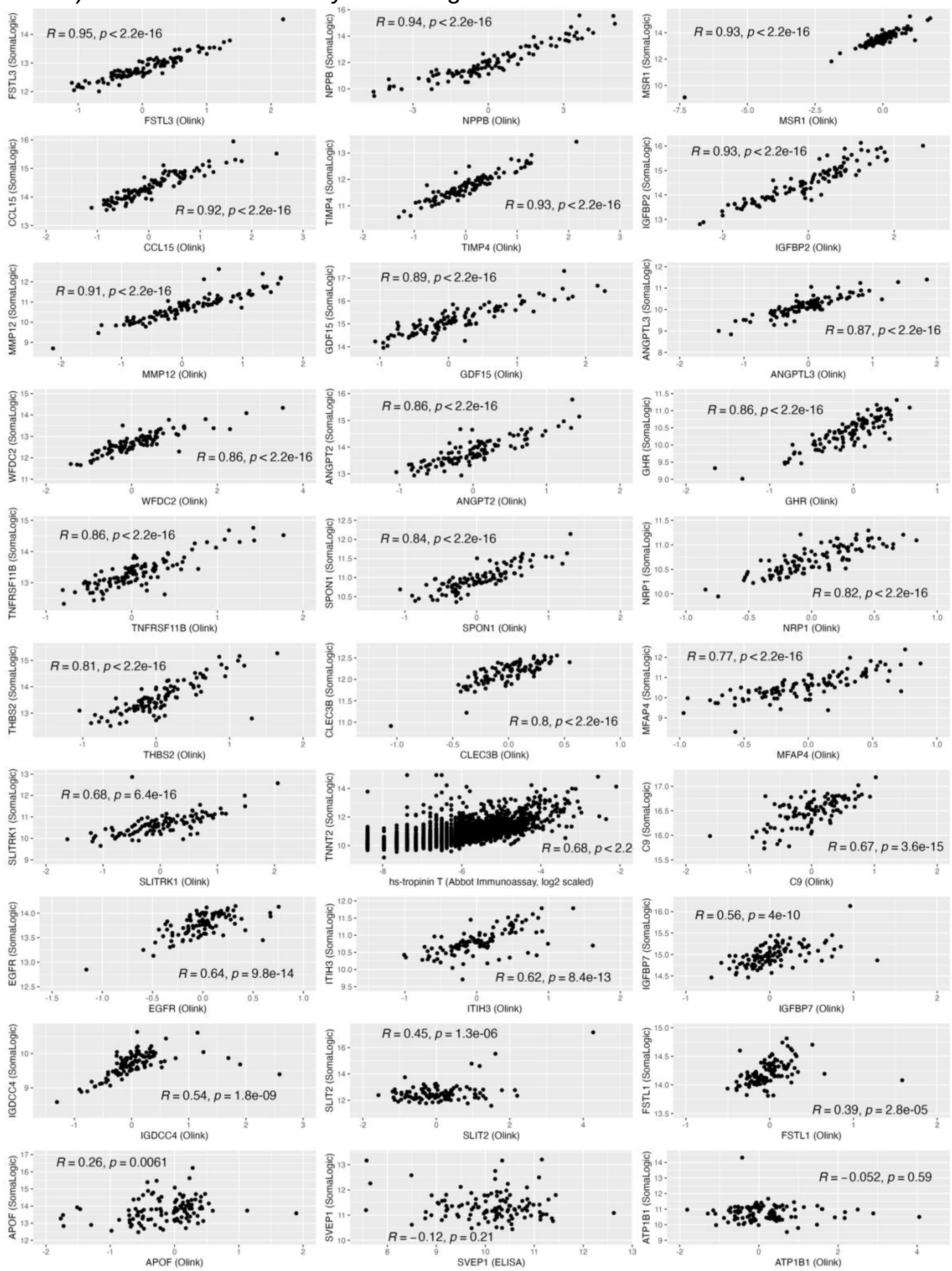

Supplementary Figure 7: GTeX heatmap of candidate protein expression levels in different types of tissues.

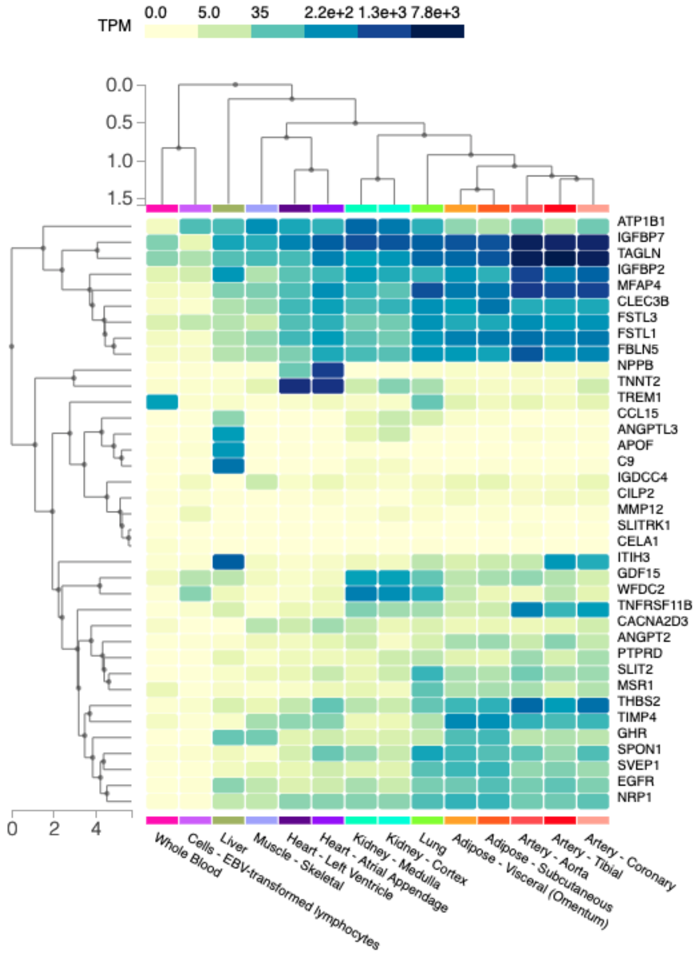

Supplementary Figure 8: Two-sample Mendelian Randomization Manhattan plots using only cis-pQTLs. Hatched line indicates nominal significance (p <0.05). Solid red line indicates significance after Bonferroni multiple testing correction. Gray pQTLs – non-significant, Blue – nominally significant, Red – significant after multiple testing correction. pQTLs were obtained from the INTERVAL (n=3,301), AGES (n=5,368), and Fenland (n=10,708) studies. The summary statistics for HF were obtained from the HERMES consortium (n=977,323). Summary statistics for atrial fibrillation were obtained from a GWAS meta-analysis of 6 studies (n = 1,030,836), for CHD were from UK Biobank and replicated using CARDIoGRAMplusC4D data (n = 296,525), for CKD were from a 43 study GWAS meta-analysis (n = 117,165), for DM were from a GWAS meta-analysis of 3 studies (n = 655,666), and for hypertension were from a UK Biobank GWAS (n = 463,010). Summary statistics for LVEDV, LVESV and LVEF were obtained from UK Biobank (n=36,041). See Methods. Source data are provided as a Source Data file.

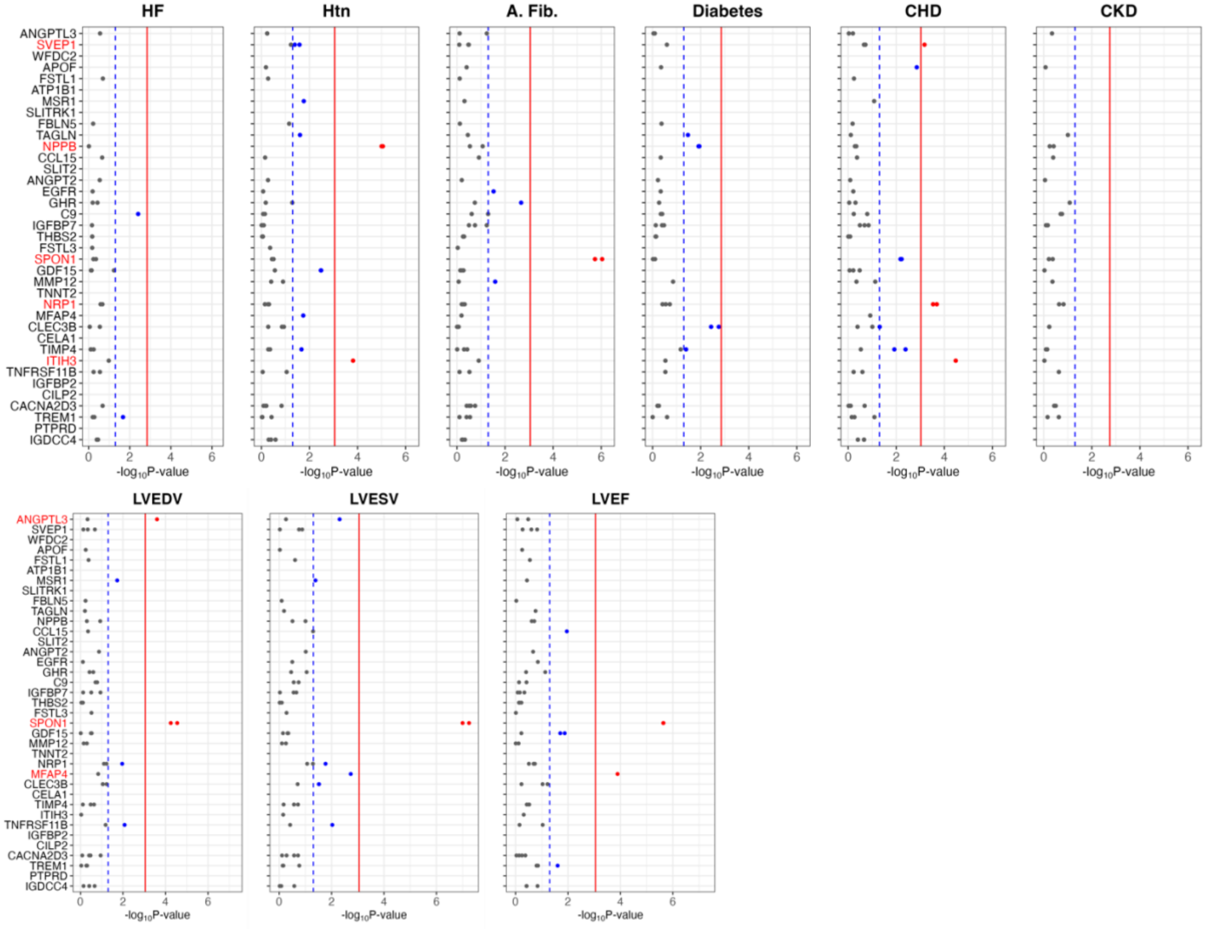

Supplementary Figure 9: GTeX violin plots showing the expression levels of protein quantitative trait loci (pQTLs) that were also found to be expression quantitative trait loci (eQTLs).

SPON1 (rs10832169)

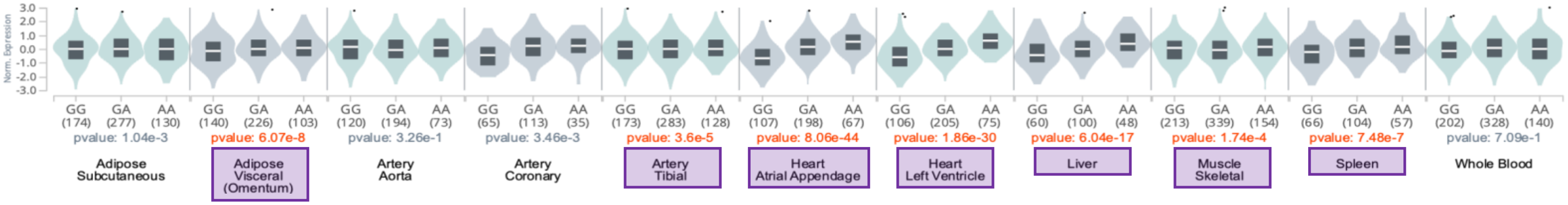

MFAP4 (rs139356332)

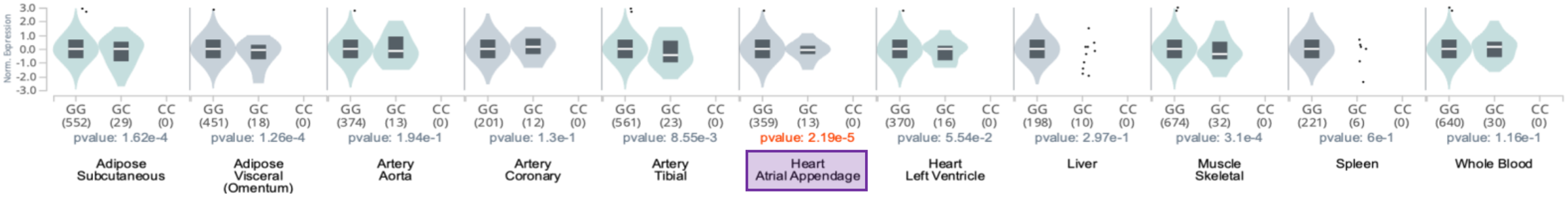

ITIH3 (rs2535629)

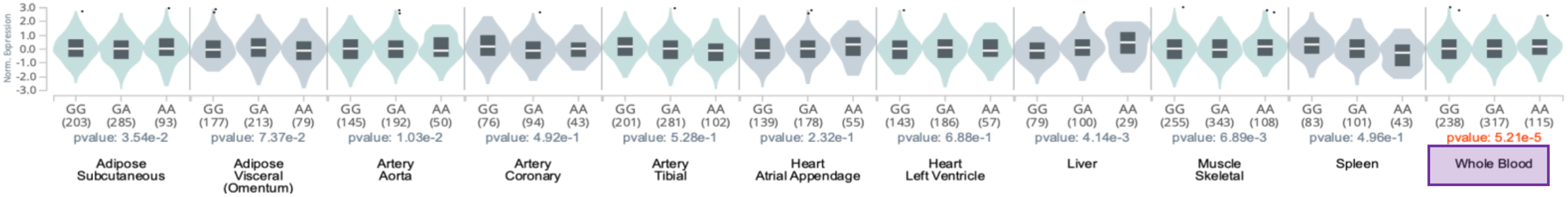

Supplementary Figure 10: Diagnostic plots for consensus clustering analysis at ARIC visit 5 (n= 4,483).

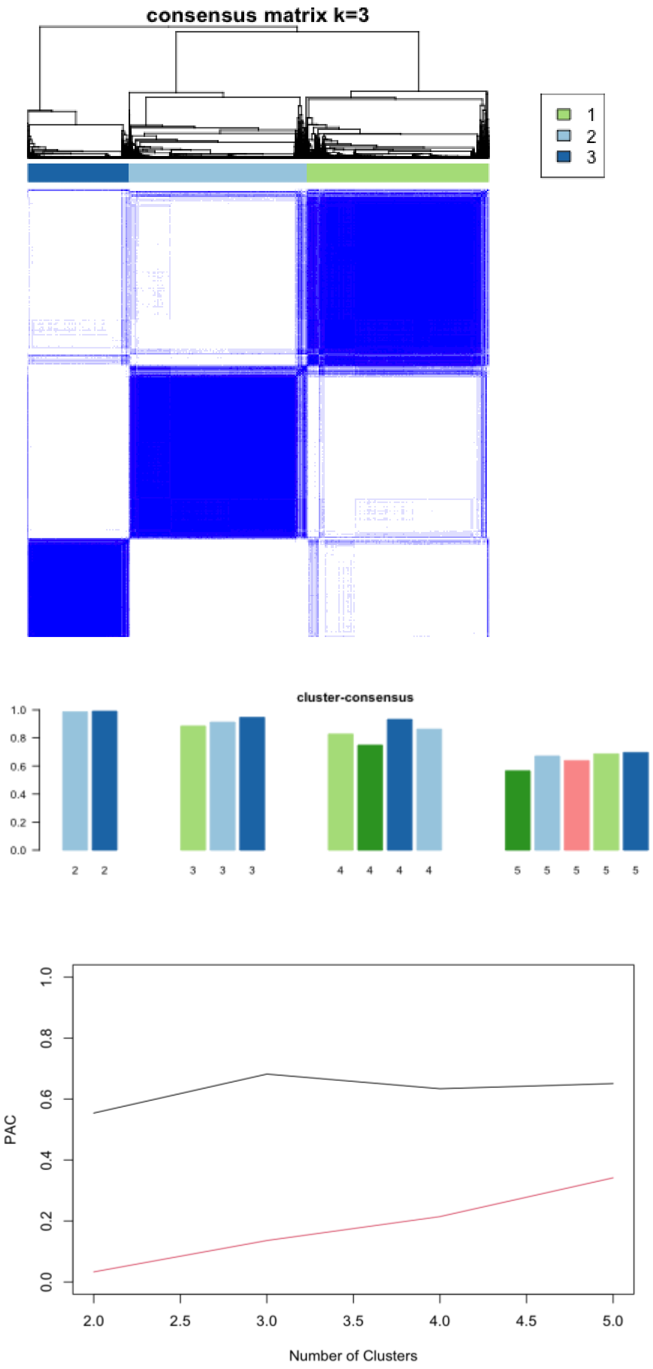

Supplement: Supplementary file 1 — Supplementary Information [file 41467_2023_44680_MOESM1_ESM.pdf]
