# Supplementary material for: Large scale plasma proteomics identifies novel proteins and protein networks associated with heart failure development
Source: Nat Commun. 2024 Jan 15;15:528. doi: 10.1038/s41467-023-44680-3 (PMC10789789; doi:10.1038/s41467-023-44680-3)
Supplement: Supplementary file 3 — Description of Additional Supplementary Files [file 41467_2023_44680_MOESM3_ESM.pdf]

**Title:** Supplementary Data 1.
**Description:** Top enriched pathways from IPA analysis for the set of proteins associated with HF at Bonferroni significance in meta-analysis of ARIC Vist 3, ARIC Visit 5, and HUNT data. Fisher's exact test was used to determine overrepresentation.

**Title:** Supplementary Data 2.
**Description:** ARIC cis pQTLs for candidate proteins.

**Title:** Supplementary Data 3.
**Description:** Annotation of candidate proteins using data from the Human Protein Atlas (HPA).

**Title:** Supplementary Data 4.
**Description:** pQTLs identified for the key heart failure (HF) associated proteins.

**Title:** Supplementary Data 5.
**Description:** Single SNP MR results that replicate in multiple distinct datasets.

**Title:** Supplementary Data 6.
**Description:** Results of bidirectional MR for significant multi-marker hits in preliminary MR analysis.

**Title:** Supplementary Data 7.
**Description:** Results of colocalization analysis for significant associations in Medelian randomization.

**Title:** Supplementary Data 8.
**Description:** Information on druggability of potentially causal proteins queried from The Druggable Genome manuscript.

**Title:** Supplementary Data 9.
**Description:** Results of analyses testing for association of WGCNA modules with incident heart failure in ARIC visit 3 and visit 5.

**Title:** Supplementary Data 10.
**Description:** Top enriched pathways for WGCNA Brown submodules from IPA analysis.

**Title:** Supplementary Data 11.
**Description:** Top enriched pathways for WGCNA Salmon module from IPA analysis.

**Title:** Supplementary Data 12.
**Description:** Previously described associations of candidate proteins with incident HF and clinical outcomes among patients with prevalent HF (HF overall, HFpEF, HFrEF).
