# Supplementary material for: Large scale plasma proteomics identifies novel proteins and protein networks associated with heart failure development
Source: Nat Commun. 2024 Jan 15;15:528. doi: 10.1038/s41467-023-44680-3 (PMC10789789; doi:10.1038/s41467-023-44680-3)
Supplement: Supplementary file 16 — Reporting Summary [file 41467_2023_44680_MOESM16_ESM.pdf]

nature portfolio

Corresponding author(s): Amil M. Shah, MD MPH

Last updated by author(s): Oct 12, 2023

Reporting Summary

Nature Portfolio wishes to improve the reproducibility of the work that we publish. This form provides structure for consistency and transparency in reporting. For further information on Nature Portfolio policies, see our Editorial Policies and the Editorial Policy Checklist.

Statistics

For all statistical analyses, confirm that the following items are present in the figure legend, table legend, main text, or Methods section.

n/a	Confirmed	
☐	☒	The exact sample size (*n*) for each experimental group/condition, given as a discrete number and unit of measurement
☐	☒	A statement on whether measurements were taken from distinct samples or whether the same sample was measured repeatedly
☐	☒	The statistical test(s) used AND whether they are one- or two-sided<br>*Only common tests should be described solely by name; describe more complex techniques in the Methods section.*
☐	☒	A description of all covariates tested
☐	☒	A description of any assumptions or corrections, such as tests of normality and adjustment for multiple comparisons
☐	☒	A full description of the statistical parameters including central tendency (e.g. means) or other basic estimates (e.g. regression coefficient) AND variation (e.g. standard deviation) or associated estimates of uncertainty (e.g. confidence intervals)
☐	☒	For null hypothesis testing, the test statistic (e.g. *F*, *t*, *r*) with confidence intervals, effect sizes, degrees of freedom and *P* value noted<br>*Give P values as exact values whenever suitable.*
☐	☒	For Bayesian analysis, information on the choice of priors and Markov chain Monte Carlo settings
☐	☒	For hierarchical and complex designs, identification of the appropriate level for tests and full reporting of outcomes
☒	☐	Estimates of effect sizes (e.g. Cohen's *d*, Pearson's *r*), indicating how they were calculated

Our web collection on statistics for biologists contains articles on many of the points above.

Software and code

Policy information about availability of computer code

Data collection	No software was used for data collection.
Data analysis	All analyses were conducted using R (version 4.2.0), including R packages "randomForestSRC" (version 3.1.1), "TwoSampleMR" (version 0.5.6), "coloc" (version 5.1.0), "ConsensusClusterPlus" (version 1.61.0), "WGCNA" (version 1.71). Additional pathway enrichment analysis was performed using the QIAGEN Ingenuity Pathway Analysis software (Hilden, Germany).

For manuscripts utilizing custom algorithms or software that are central to the research but not yet described in published literature, software must be made available to editors and reviewers. We strongly encourage code deposition in a community repository (e.g. GitHub). See the Nature Portfolio guidelines for submitting code & software for further information.

Data

Policy information about availability of data

All manuscripts must include a data availability statement. This statement should provide the following information, where applicable:

- Accession codes, unique identifiers, or web links for publicly available datasets
- A description of any restrictions on data availability
- For clinical datasets or third party data, please ensure that the statement adheres to our policy

Anonymized data from the ARIC study are available at the NHLBI Biologic Specimen and Data Repository Information Coordinating Center and can be accessed through the website (https://biolincc.nhlbi.nih.gov/studies/aric/). Requests for access of ARIC data may also be submitted to the ARIC Publications Committee

according to established study procedures. Additional databases and availability are as follows:
- IEU Open GWAS Project Database (https://gwas.mrcieu.ac.uk/)
-- INTERVAL pQTL data (PMID: 29875488, multiple pQTLs)
-- HERMES HF data (id:ebi-a-GCST009541)
-- AFib Data (id:ebi-a-GCST006414)
-- CHD Data (id:ebi-a-GCST005194)
-- CKD Data (id:ebi-a-GCST003374)
-- DM Data (id:ebi-a-GCST006867)
-- Htn Data (id:ukb-b-12493)

- AGES data (DOI: 10.1038/s41467-021-27850-z, Supplemental Table 3)
- Fenland data (DOI: 10.1126/science.abj1541, Supplemental Table 2)
- UK Biobank CMR phenotypes (Broad Institute
Cardiovascular Disease Knowledge Portal under the 'Downloads' tab at http://www.
broadcvdi.org/)
- IPA and QIAGEN Knowledge base
-GTeX (www.gtexportal.org, dbGaP Accession phs000424.v8.p2)
- Human Protein Atlas (www.proteinatlas.org)
- Druggable genome (DOI: 10.1126/scitranslmed.aag1166, Table S1)
- ChEMBL (www.ebi.ac.uk/chembl/)

Research involving human participants, their data, or biological material

Policy information about studies with human participants or human data. See also policy information about sex, gender (identity/presentation), and sexual orientation and race, ethnicity and racism.

Reporting on sex and gender	Gender was ascertained by participant self-report at all ARIC study visits. All statistical models adjusted for gender. Stratified analysis by gender was not performed.
Reporting on race, ethnicity, or other socially relevant groupings	Race and ethnicity were ascertained by participant self-report at all ARIC study visits. All statistical models adjusted for race/ethnicity. Stratified analysis by race/ethnicity was not performed.
Population characteristics	Characteristics of the 10,638 heart failure-free ARIC participants at study Visit 3 included age 60±5 years, 54% women, 21% Black race. Characteristics of the 4,483 HF-free ARIC participants at study Visit 5 included age 75±5 years, 58% women, 17% Black race). Characteristics of the 3,262 HUNT participants from the third study cycle included age 65±10 years, 39% women, 0% Black race).
Recruitment	The Atherosclerosis Risk in Communities (ARIC) study is a prospective epidemiologic cohort study that enrolled 15,792 participants between 1987 and 1989 at 4 communities in the United States: Forsyth County, NC, Jackson, MS, suburban Minneapolis, MN, and Washington County, MD. Participants underwent four exam visits between 1987 and 1998, followed by a fifth exam visit between 2011 and 2013. The mid-life baseline analysis set in this analysis used data from ARIC participants attending the third study visit which occurred between 1993-1995 who were free of heart failure (HF) at the time of study visit. The late-life baseline analysis set in this analysis used data from ARIC participants attending the fifth study visit which (2011-2013) who were free of HF at the time of the study visit. The Trgndelag Health Study (HUNT) Study is a population-based cohort study that collected detailed socio-demographic and clinical information for 229,000 participants from Trondelag County in Norway. This study included 3,262 individuals from the third survey (HUNT 3) which enrolled a total of 50,807 participants between 2006-2008.
Ethics oversight	In ARIC, The study protocol was approved by institutional review boards at each field center: University of North Carolina at Chapel Hill, Chapel Hill, NC; Wake Forest University, Winston-Salem, NC; Johns Hopkins University, Baltimore, MD; University of Minnesota, Minneapolis, MN; and University of Mississippi Medical Center, Jackson, MS. All participants provided written informed consent at each study visit. In HUNT, All individuals provided informed written consent and the study was approved by the Regional Committee for Medical and Health Research Ethics (REK South-East C 2019/17355).

Note that full information on the approval of the study protocol must also be provided in the manuscript.

Field-specific reporting

Please select the one below that is the best fit for your research. If you are not sure, read the appropriate sections before making your selection.

[x] Life sciences   [ ] Behavioural & social sciences   [ ] Ecological, evolutionary & environmental sciences

For a reference copy of the document with all sections, see nature.com/documents/nr-reporting-summary-flat.pdf

Life sciences study design

All studies must disclose on these points even when the disclosure is negative.

Sample size	No a priori sample size calculations were performed as this was an an analysis of an ongoing observational cohort study.. The sample size was chosen based on the number of participants in the ARIC and HUNT cohort studies with available proteomic data and information on prevalent and incident heart failure events. This approach was taken to maximize sample size and study power. The power was deemed to be adequate as prior studies of similar design but smaller sample sizes were able to detect significant associations between circulating proteins and heart failure risk..
Data exclusions	ARIC and HUNT participants with prevalent heart failure at the time of sample collection for proteomics measures were excluded. Participants who did not have proteomics measured were excluded.
Replication	We performed a parallel analysis in two independent cohorts (ARIC, HUNT) to ensure the reproducibility and generalizability of our findings. As detailed in the manuscript results, not all proteins identified in one cohort were also identified in the other cohort.
Randomization	This was an observational analysis, not a randomized trial. To help control for confounding, analyses of associations between plasma proteins and outcomes employed multivariable regression models that adjusted for established heart failure risk factors.
Blinding	Blinding was not applicable in this study as this was an observational analysis, not a randomized trial.

Reporting for specific materials, systems and methods

We require information from authors about some types of materials, experimental systems and methods used in many studies. Here, indicate whether each material, system or method listed is relevant to your study. If you are not sure if a list item applies to your research, read the appropriate section before selecting a response.

Materials & experimental systems

n/a	Involved in the study
☒	☐ Antibodies
☒	☐ Eukaryotic cell lines
☒	☐ Palaeontology and archaeology
☒	☐ Animals and other organisms
☒	☐ Clinical data
☒	☐ Dual use research of concern
☒	☐ Plants

Methods

n/a	Involved in the study
☒	☐ ChIP-seq
☒	☐ Flow cytometry
☒	☐ MRI-based neuroimaging

Plants

Seed stocks	*Report on the source of all seed stocks or other plant material used. If applicable, state the seed stock centre and catalogue number. If plant specimens were collected from the field, describe the collection location, date and sampling procedures.*
Novel plant genotypes	*Describe the methods by which all novel plant genotypes were produced. This includes those generated by transgenic approaches, gene editing, chemical/radiation-based mutagenesis and hybridization. For transgenic lines, describe the transformation method, the number of independent lines analyzed and the generation upon which experiments were performed. For gene-edited lines, describe the editor used, the endogenous sequence targeted for editing, the targeting guide RNA sequence (if applicable) and how the editor was applied.*
Authentication	*Describe any authentication procedures for each seed stock used or novel genotype generated. Describe any experiments used to assess the effect of a mutation and, where applicable, how potential secondary effects (e.g. second site T-DNA insertions, mosiacism, off-target gene editing) were examined.*
